# Microbial Production of Ethanol From Sludge Derived From an Urban Wastewater Treatment Plant

**DOI:** 10.3389/fmicb.2018.02634

**Published:** 2018-11-01

**Authors:** Patricia Godoy, Álvaro Mourenza, Sergio Hernández-Romero, Jesús González-López, Maximino Manzanera

**Affiliations:** Department of Microbiology, Institute for Water Research, University of Granada, Granada, Spain

**Keywords:** microethanol, wastewater sludge, proteus, biofuel, wastewater treatment, residue valorization

## Abstract

A collection of lipase-producing microorganisms was isolated from sludge derived from an urban wastewater treatment plant. The microorganisms with the highest levels of lipase activity were selected in order to use triglycerides present in the sludge effectively and were then transformed with *pdc:adhB* genes for the production of ethanol. The transgenic strains showed high growth rates in diluted sludge and produced lipase protein in order to utilize fat present in the sludge, which provides an abundant source of carbon. Using sludge derived from treated wastewater as nutrient source, ethanol was produced by certain transgenic species belonging to the genera *Proteus*. Different forms of sludge were tested for maximal ethanol production, with dehydrated sludge being found to produce the best performance.

## Introduction

Wastewater treatment generates large quantities of sewage sludge, which is estimated to account for ~1–2% of the total volume of treated wastewater. Some studies estimate that Europe, USA and China together generate 240 million wet tons of sludge per year from treated wastewater (Pritchard et al., 2010). The removal of these enormous quantities of sludge generated is a costly exercise for water management companies. This sludge, produced from chemically and biologically treated wastewater in sewage treatment plants, contains a large number of microorganisms and a wide range of organic and inorganic substances. The microbial composition of sludge, which has been studied extensively, has been shown to always contain the same types of microorganism for the specific activities required during the wastewater decontamination process (Rodriguez et al., 2015; Saunders et al., 2015; Gonzalez-Martinez et al., 2016).

Wastewater sludge is composed of water, organic compounds, macro- and micro-nutrients and trace elements which microorganisms can use as nutrient sources. Although sludge composition depends on the type of wastewater treatment used, organic materials generally account for up to 50% of the total, of which 17–30% of dry weight is in the form of fat (Martinez-Toledo et al., 2012). This fat is produced by direct absorption of lipids present in the wastewater by sludge particles and phospholipids released by the cell membranes of microbiota (mainly bacteria); in addition, fat comes from metabolites and cell lysis by-products (Jarde et al., 2005). The sludge bacteria adapt to this environment and survive by consuming nutrients present in the sludge. Indigenous microorganisms from the sludge are better suited to using sludge as nutrient source than microorganisms imported from other environments.

The term microdiesel was coined by Kalscheuer et al. (2006) to describe diesel produced in the form of fatty acid methyl esters (FAMEs) and fatty acid ethyl esters (FAEEs) by *Escherichia coli* from glucose and oleic acid. This is caused by the insertion of genes coding for ethanolic enzymes pyruvate decarboxylase (Pdc) and alcohol dehydrogenase (AdhB) from *Zymomonas mobilis*, as well as unspecific acyltransferase WS/DGAT from *Acinetobacter baylyi* strain ADP1. However, these potential carbon sources are too expensive for industrial applications. Recently, a study reviewed various ways of producing microdiesel and focused on different strains which accumulate fats and supply carbon to microorganisms (Bhatia et al., 2017). The authors suggest using microdiesel, obtained from microbes and renewable materials as carbon sources, as an alternative to petroleum diesel. These microorganisms can, in turn, be engineered to accumulate and transform fatty acid content into ethanol, FAMEs or FAEEs. To our knowledge, this is the first time that wastewater sludge has been proposed as a C source for ethanol production through the use of modified microorganisms isolated from wastewater sludge. We also propose a new way of using this type of residue to reduce the negative impact of sludge derived from treated wastewater.

## Materials and methods

### Samples collection and isolation of lipolytic bacteria using a standard method

One liter of either digested or non-digested, non-skimmed sludge and fat produced by skimming sludge from the Vados wastewater treatment plant in Granada (Spain) (37.11 N; 3.4 W) was collected and stored at 4°C for later use. One gram from each sample was serially diluted in saline buffer, and a 100-μl aliquot from each dilution was plated on tributyrin agar (TBA) plates.

The TBA plates contained the following items (%): 0.5 peptone, 0.3 yeast extract, 1 agar and 0.1 ml tributyrin. The pH was adjusted to 5.5. Membrane filtration (Type HA 0.45 lm Schott Mainz, Germany) was used to sterilize tributyrin, and the filtrate was added to the base growth medium.

After 24 h of incubation at 30°C, individual colonies were selected and streaked out to obtain pure cultures based on the extent of the enzyme diffusion zone after incubation depending on their lipolytic activity according to the method described by Sztajer et al. (1988). Tests were performed in triplicate.

### 16S rRNA gene sequencing and phylogenetic analysis

The isolated selected strains were identified by analyzing the partial sequence of the gene encoding 16S rRNA. The primers fD1, fD2, rD1, and rD2 were synthesized by Sigma Genosys Ltd. (U.K.) and then used to amplify and sequence virtually the full length of the 16S rRNA gene according to the method described by Weisburg et al. (1991).

### Lipase activity

Lipolytic activity was determined using *p*-nitrophenil palmitate (pNPP) as substrate as described by Gupta et al. (2002) and Ertugrul et al. (2007). Briefly, 1 ml cell-free culture was mixed with 1 ml substrate solution and incubated at 30°C for 30 min. The reaction was interrupted by a 4-min incubation at 100°C. Absorbance was then recorded at a wavelength of 410 nm using a Hitachi U-200 spectrophotometer. A substrate solution was freshly made by mixing 10 ml of solution A (30 mg pNPP in 10 ml isopropanol) with 90 ml solution B (0.1 g Arabic gum; 0.4 ml Triton X-100 in 90 ml 50 mM Tris-HCl pH8). Increasing concentrations of commercial lipase from *Burkholderia cepacia* (ref. 62309; from Sigma-Aldrich) were used for the calibration curve.

### Construction of ethanol-producing strains

To construct the ethanol-producing strains, pyruvate decarboxylase (*pdc*) and alcohol dehydrogenase (*adh*) genes from *Z. mobilis* were inserted into the lipase-producing strains with the aid of a mini-transposon harbored in a pSEVA_234_pdc:adh plasmid kindly supplied by Dr. de Lorenzo (Nikel and de Lorenzo, 2013). The plasmid was transferred by tetraparental mating with *E. coli* CC118λpir as the donor strain and two helper strains *E. coli* HB101 (pRK600) and *E. coli* CC118λpir (pBEXK) (Nikel and de Lorenzo, 2013). All strains were grown on trypticase soy broth (TSB) with a selective antibiotic, 1 ml from each culture was centrifuged at 5,000 rpm for 5 min and the antibiotic was washed away. The different strains were then incubated on a sterile filter placed on a TSA plate for 24 h at 30°C according to the method described by Choi and Schweizer (2006).

### Stability assays of genetic markers in lipolytic strains

To analyze ethanol production by the recombinant microbial strains, the stability of the transposon used in *Proteus sp*. strains was tested in the absence of antibiotic. Briefly, the recombinant strains were cultured overnight in tryptic soy broth (TSB) medium supplemented with kanamycin (50 μg ml^−1^) at 37°C in an orbital shaker at 200 rpm. Subsequently, 200 μl from each culture was spread on PDA plates and incubated at 37°C for 5 h until a thin growth film became visible. Biomass from each strain was resuspended in 1 ml of M9 medium, and serial dilutions were plated onto TSA plates with and without kanamycin (50 μg ml^−1^). Plasmid stability was determined by calculating the ratio between CFU ml^−1^ in the presence and absence of antibiotic.

### Ethanol determination

The commercial NZY Gene and Enzyme Kit (Ref. AK00061) was used to determine ethanol produced by the different microorganisms according to the protocol provided by the supplier. This method involves spectrophotometrically measuring NADH produced by reactions after alcohol dehydrogenase (ADH) and aldehyde dehydrogenase (AlDH) are added.

### Statistical analyses

For statistical testing purposes, the Student's *t*-test was implemented in SPSS 15.0 software (IBM Corporation). *P* ≤ 0.05 was chosen as the cut-off point for statistical significance.

## Results and discussion

### Isolation of lipase-producing microorganisms

Indigenous microorganisms are normally best suited to performing and maximizing specific metabolic functions in particular environments. As fat is regarded as the most energetic fraction of wastewater sludge for biofuel production, we decided to isolate lipase-producing microorganisms from four different types of sludge produced by the wastewater treatment plant in Granada (Spain) depending on their capacity to use fat as carbon source.

**Figure 1:**
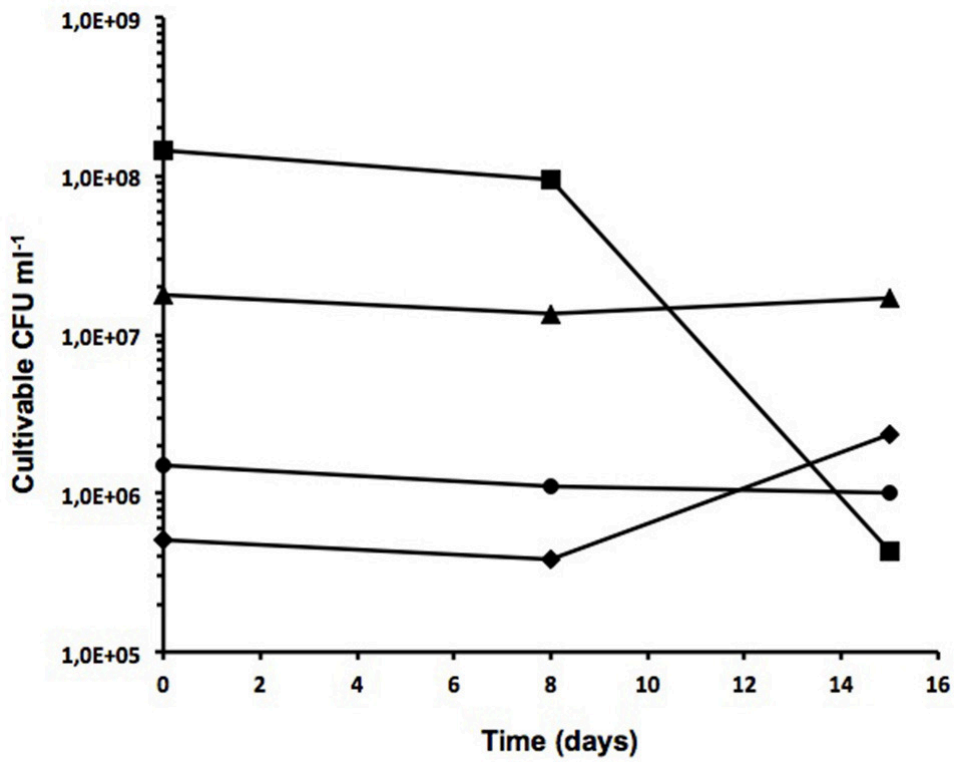
Cultivable cells from different treated wastewater fractions. Number of cultivable cells in TSA from non-treated sludge (triangles), skimmed sludge (diamonds), fat from skimmed sludge (squares), and digested sludge (circles). Sampling time is indicated in days.

These included non-treated sludge (NS), skimmed sludge (SS), fat from skimmed sludge (F) and digested sludge (DS). One liter of sludge from each sample was incubated at room temperature for 15 days. By plating serial dilutions on standard TSA plates at three different sampling times (0, 8 and 15 days), the number of cultivable microorganisms was determined to be approximately 10^6^ CFU ml^−1^ in all fractions at time 0 with some slight variations (Figure 1). Greater numbers of cultivable bacteria were observed at time 0 for samples F and NS (~144 × 10^6^ and 18 × 10^6^ CFU ml^−1^, respectively), indicating a larger number of cultivable microorganisms at higher nutrient concentrations. Although cultivable microorganisms remained relatively constant in NS samples, an almost 2-fold reduction in the number of cultivable cells was observed over a 15-day period in sample F. With regard to DS samples, the number of cultivable cells remained quite stable at around 1 × 10^6^ CFU ml^−1^ over the 15-day period of analysis. However, a slight increase from 0.5 × 10^6^ to 2.4 × 10^6^ CFU ml^−1^ in the number of cultivable cells was observed in SS samples over the same period.

**Figure 2:**
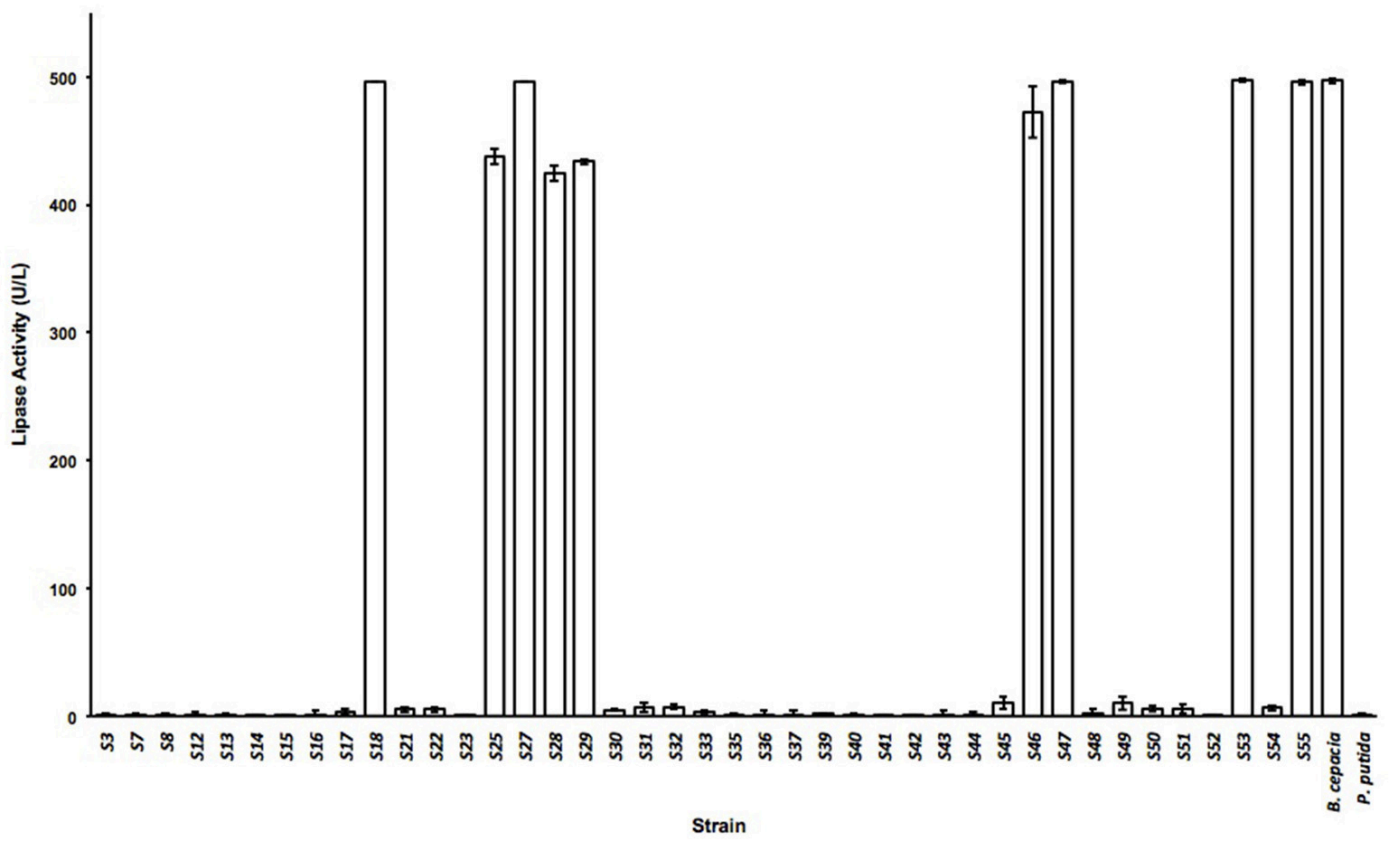
Lipase activity. Activity of supernatants from the different microbial isolates as units of lipase per liter of supernatant. Error bars show the standard deviations for at least three replicates.

TBA plates were used in parallel to detect colonies with lipolytic activity at the specified sampling times from the same samples as described in section Material and Methods. A total of 12 different isolates were identified in NS samples, fourteen in SS samples, nine different strains in DS samples, and a further nine strains in F samples. The nature of the different isolates was determined according to their capacity to grow on 0.25 M maltose, glycerol, arabinose and glucose and according to their catalase activity or ability to survive after a 30-min incubation period at 72°C due to the presence of spores (data not shown). Lipase tests of culture supernatants from the various isolates were carried out using the well-characterized lipase-producing strain *Burkholderia cepacia* as reference. On the basis of these tests, 5 different isolates were selected which showed lipase activity values similar to those for *B. cepacia* (Figure 2) as they reached the maximal possible value by this method. These isolates were taxonomically assigned to species from the genera *Microbacterium* (S18), *Acinetobacter* (S27), and *Proteus* (S47; S53; and S55). This coincides with previous descriptions of lipases isolated from *Microbacterium* sp. (Jaeger and Eggert, 2002; Joseph et al., 2012; Tripathi et al., 2014), *Acinetobacter* sp. (Ahmed et al., 2010; Uttatree et al., 2010; Cherif et al., 2011; Zheng et al., 2011) and *Proteus* sp. (Tanasupawat et al., 2016; Gupta et al., 2018). These strains were isolated from DS (*Microbacterium* sp. S18 with GenBank Accession Numbers MH800317), NS (*Acinetobacte*r sp. S27 and *Proteus* sp. S47 with GenBank Accession Numbers MH800318 and MH800319, respectively) and SS samples (*Proteus* sp. S53, and *Proteus* sp. S55 with GenBank Accession Numbers MH800320 and MH8000321, respectively).

### Genetic amenability of isolates

After lipase-producing microorganisms from the wastewater treatment plant were selected due to their particularly high lipase activity levels, genetic information required for ethanol production was inserted. As most strains isolated from the natural environment are not amenable to genetic modification, we first tested their ability to receive exogenous genetic markers, whose genomic stability was also evaluated. We used mini-Tn7 technology developed by Dr. de Lorenzo's laboratory to first assess the capacity of strains to be transformed by plasmid pUX-BF13 which bears the *gfp* gene coding for the green fluorescent protein (Nikel and de Lorenzo, 2013). Prior to mini-Tn7 conjugation, antibiotic-resistant strains were obtained by incubating the different isolates with increasing antibiotic concentrations. *Proteus* sp. S53 and *Proteus* sp. S47 rifampicin mutants (S53R and S47R, respectively), as well as a *Proteus* sp. S55 tetracycline mutant (S55T) and an *Acinetobacter* sp. S27 streptomycin mutant (S27S) were obtained.

For the fluorescent versions of the antibiotic-resistant strains, tetraparental mating, as described in Materials and Methods, was performed using *E. coli* GFP_2_ as donor strain, *E. coli* HB101 (pRK600) and *E. coli* (pUX-BF13) as helper strains, as well as S53R, S47R, S55T, and S27S as recipient strains. To select the transconjugant strains we used plates containing TSA with gentamicin (Gm10 μg ml^−1^). Fluorescent strains were identified and selected using UV light irradiation.

**Figure 3:**
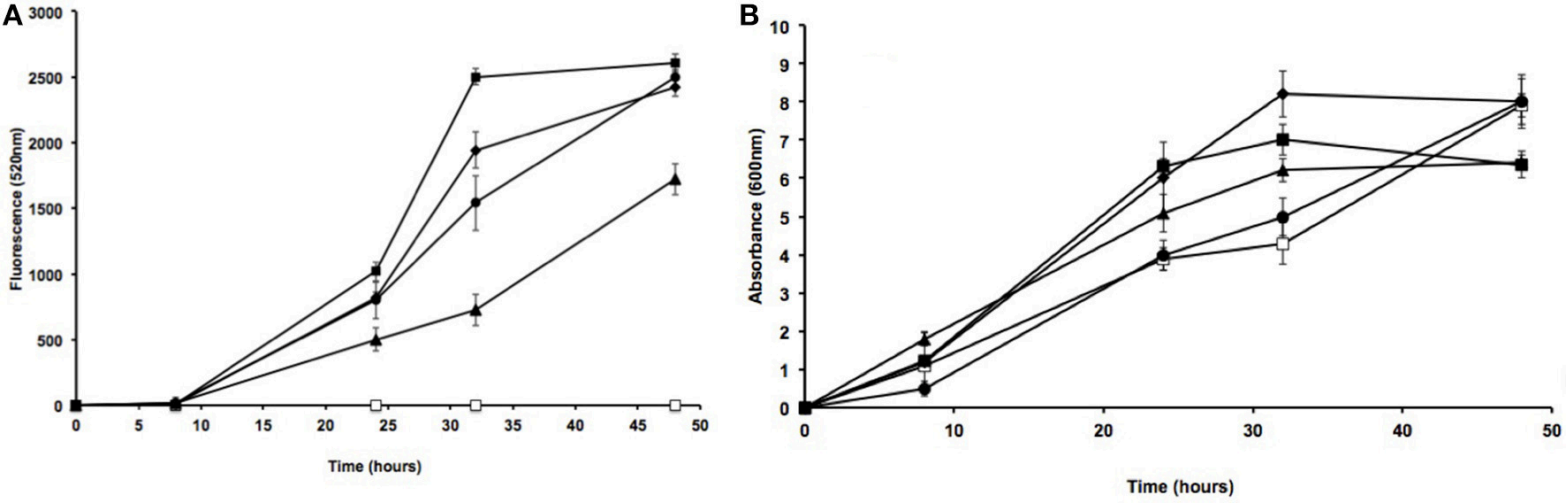
Fluorescence and absorbance of *gfp*-modified strains. Fluorescence emission (520 nm) **(A)** and growth (as absorbance at 600 nm) **(B)** by different lipase-producing isolates were measured at different times. Strains: S27 (open squares), S47 (solid squares), S53 (solid triangles), S55 (solid diamonds), *E. coli* GFP2 (solid circles). Error bars show the standard deviations for at least three replicates.

To determine the stability of inserts from the different isolates, the fluorescent mutant strains were cultivated in TSB for over 48 h at 180 rpm and at 30°C in the absence of antibiotics. Strain fluorescence, which was recorded at 0, 8, 24, 32, and 48 h at 520 nm using a fluorometer, was compared to culture absorbance at 600 nm by spectrophotometry (Figure 3). We observed an increase in fluorescent emission with the culture growth for the four different strains except *Acinetobacter* sp. S27S which showed no florescence despite its ability to grow on TSB media. This could be explained by the absence of the attTn7 insertion site in *Acinetobacter* strains, which has been described for other *Proteus* strains such as *Proteus mirabilis* HI4320. It is important to note that the presence of this insertion site significantly increases conjugation efficiency (Choi and Schweizer, 2006).

### Introduction of PDC- and ADH-genes into lipase-producing strains generates ethanol

**Figure 4:**
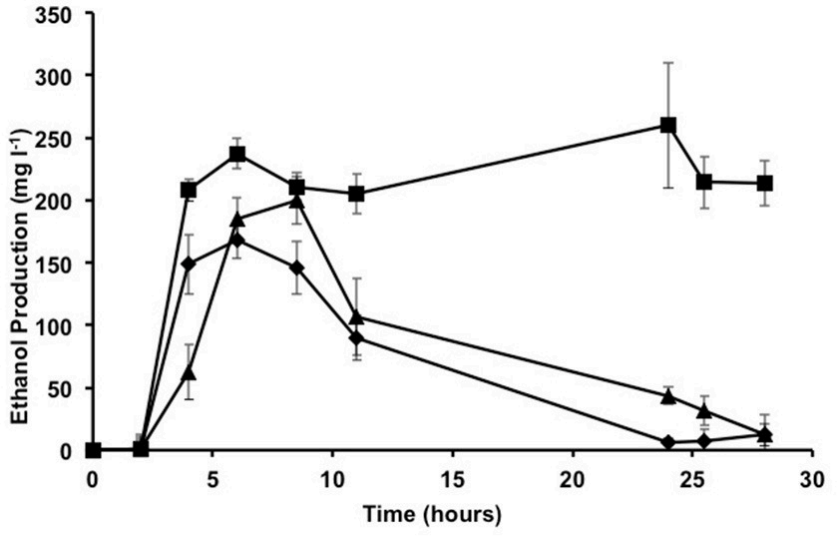
Ethanol production in TSB. Amount of ethanol detected in the culture supernatants of *Proteus* sp. S47Rpdcadh (squares), *Proteus* sp. S53pdcadh (triangles) and *Proteus* sp. S55pdcadh (diamonds) in TSB. Error bars show the standard deviations for at least three replicates.

After demonstrating the genetic amenability of *Proteus* strains and the genetic stability of inserts, the *pdc* and *adh* genes from *Z. mobilis* were transferred to *Proteus* sp. S53R, S47R, and S55T strains using a method similar to that described above. The resulting strains were named *Proteus* sp. S53Rpdcadh, S47Rpdcadh and S55Tpdcadh due to the presence of *pdc* and *adh* genes and given specific antibiotic resistance. The ability to produce ethanol upon insertion of these genes was tested using the Nzytech Ethanol Kit (Ref AK00061) after growing the strains in TSB. Growth in TSB followed a standard pattern, with exponential growth being reached 6 h after inoculation (initial absorbance of 0.05 at 600 nm) with an absorbance of ~3, followed by a stationary phase of at least 28 h (data not shown). *Proteus* sp. S47Rpdcadh produced ~230 mg l^−1^ of ethanol after 6 h when grown in TSB (Figure 4), which remained at a similar level for the rest of the assay period. In the case of *Proteus* sp. S53Rpdcadh, the amount of ethanol produced was slightly lower, reaching a similar level at 8 h, but then fell sharply. Ethanol produced by *Proteus* S55Tpdcadh reached a maximum level (168 mg l^−1^) at 6 h, whose concentration then decreased gradually. As expected, parental strains without *pdc* or *adh* genes produced no ethanol (data not shown).

### Ethanol production using wastewater sludge as substrate

**Figure 5:**
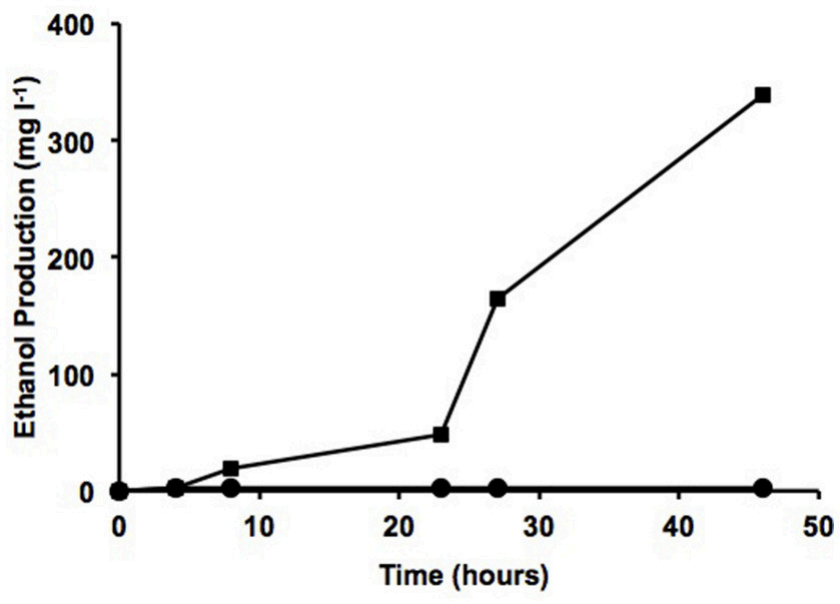
Ethanol production from digested sludge. Production of ethanol (mg l^−1^) by *Proteus* sp. S47Rpdcadh from dehydrated sludge (3% w/v) in water with (squares) and without (circles) 10 g l^−1^ molasses.

Previous tests carried out in our laboratory showed that the ubiquitous bacterium *Pseudomonas putida* KT2440 is unable to survive in wastewater sludge despite its flexible and robust metabolism (data not shown) (Manzanera et al., 2001; Vilchez et al., 2008). To determine whether the isolated strains are able to survive and to produce ethanol from this stringent nutrient source, two different types of sludge from the wastewater treatment plant at Jumilla in Murcia (Spain) were used: dehydrated sludge containing over 90% dry material and pastry sludge containing 45% dry material. Water was added to the dehydrated sludge (w/w) to reach 95% water content, with both types of sample now having similar water content. Roughly 10^9^ cells from each transgenic microorganism were added to 50 ml of both types of sludge and incubated for 70 h at 30°C and 180 rpm. To monitor growth of the microorganisms added, the aliquot fractions were plated in antibiotic on TSA plates at 24, 48, and 72 h, and ethanol production was also determined at these sampling times. The *Proteus* sp. S53Rpdcadh strain was found to a have a higher survival rate, although the *Proteus* sp. S47Rpdcadh and S55Tpdcadh strains also managed to survive in both types of sludge, with over 10^7^ CFU ml^−1^ after 72 h in pastry sludge and over 10^6^ CFU ml^−1^ in dehydrated sludge. In addition to the lower growth observed in dehydrated sludge, ethanol production was also quite low in the media containing dehydrated sludge. However, the addition of another residue such as molasses sharply increased ethanol production (339 mg l^−1^) to a level even higher than that observed in TSB, thus suggesting that an alternative method for producing biofuel from residues is feasible (Figure 5). The presence of molasses alone did not result in ethanol production and other preliminary results using sludge with low concentration of fats resulted in much lower ethanol production (data not shown) pointing to the fact that the production of high concentration of ethanol requires a specific type of sludge.

Although the use of sludge to produce biofuel is not new, the literature mainly focuses on methane and biohydrogen (Castellano-Hinojosa et al., 2018). Our study shows that bio-ethanol production, based on the microbial processing of urban sludge, could be managed effectively and reutilized. Thus, bio-ethanol production might be an alternative to using the lipidic fraction (17 to 30% of the dry weight) of sludge produced by urban wastewater treatment plants. Moreover, the treatment of these residues by microbial or enzymatic catalysis could be regarded as economically advantageous, as the cost of microbial processes would be offset by low residue costs or even by waste reduction and management incentives.

Before applying these innovative processes on an mass scale, a whole set of technical and biotechnological improvements, such as the design of bio-ethanol production plants, bio-ethanol purification and further genetic engineering studies, would need to be introduced.

## Conclusions

We propose that genetically modified indigenous microorganisms be used to produce significant amounts of ethanol from waste such as dehydrated sludge and molasses. Using dehydrated sludge as substrate for the production of ethanol also helps to reduce the amount of this difficult-to-treat residue.

## Author contributions

All author listed have made a substantial, direct and intellectual contribution to the work, and approved it for publication.

### Conflict of interest statement

The authors declare that the research was conducted in the absence of any commercial or financial relationships that could be construed as a potential conflict of interest.
